# Supplementary material for: Whole-Genome Sequencing of Corallococcus sp. Strain EGB Reveals the Genetic Determinants Linking Taxonomy and Predatory Behavior
Source: Genes (Basel). 2021 Sep 15;12(9):1421. doi: 10.3390/genes12091421 (PMC8466578; doi:10.3390/genes12091421)
Supplement: Supplementary file 1 [file genes-12-01421-s001.zip › genes-1372765-supplementary/Supplemental Table S2.pdf]

Table S2. Proteins of secretion systems encoded by the strain EGB genome.

EGB gene	Component	Function and comments
**Sec system and Sec-depedent systems**		
EGBGL002418	SecA	Protein translocase, ATPase
EGBGL003236	SecY	Protein translocase
EGBGL004931	SecE	Protein translocase
EGBGL005154	SecG	Protein translocase
EGBGL002966	SecD	SecYEG protein translocase auxillary subunit
EGBGL002968	SecF	SecYEG protein translocase auxillary subunit
EGBGL002965	YajC	SecYEG protein translocase auxillary subunit
EGBGL002846	Ffh	Signal recognition particle
EGBGL006143	FtsY	Signal recognition particle-docking protein
EGBGL007913	YidC	Membrane protein insertase
**Tat system**		
EGBGL006356	TatB	Protein translocase
EGBGL006355	TatC	Protein translocase
EGBGL005048	TatA	Protein translocase
**Type II secretion system**		
EGBGL005501	GspL	Integral cytoplasmic membrane protein
EGBGL005500	GspK	Minor pseudopilin
EGBGL005499	GspJ	Minor pseudopilin
EGBGL005498	GspI	Minor pseudopilin
EGBGL005497	GspH	Minor pseudopilin
EGBGL005496	GspG	Minor pseudopilin
EGBGL005495	GspG	Minor pseudopilin
EGBGL005493	GspF	Integral cytoplasmic membrane protein
EGBGL005492	GspE	Secretion ATPase
EGBGL005491	GspD	Outer membrane secretin
EGBGL005490	GspC	Integral cytoplasmic membrane protein
EGBGL004905	GspO	Prepilin peptidase
EGBGL004904	GspD	Outer membrane secretin
EGBGL004903	GspE	Secretion ATPase
EGBGL003343	GspC	Integral cytoplasmic membrane protein
EGBGL005295	GspE	Secretion ATPase
EGBGL005495	GspG	Major pseudopilin
EGBGL005492	GspE	Secretion ATPase
**Type III secretion system**		
EGBGL006058/EGBGL005532	LcrD/YscV	Basal structure, cytoplasmic membrane protein
EGBGL006059/EGBGL005544	YscU	Basal structure, cytoplasmic membrane protein
EGBGL006060/EGBGL005545	YscT	Basal structure, cytoplasmic membrane protein
EGBGL006061/EGBGL005546	YscS	Basal structure, cytoplasmic membrane protein
EGBGL006062/EGBGL005547	YscR	Basal structure, cytoplasmic membrane protein
EGBGL006063		Hypothetical
EGBGL006064/EGBGL005549	YscQ	Likely makes up cytoplasmic C-ring
EGBGL006065		Hypothetical
EGBGL006066/EGBGL005555	YscJ	Lipoprotein in cytoplasmic membrane; component of MS ring
EGBGL006067/EGBGL005556	YscI	Hypothetical
EGBGL006068/EGBGL005560		Hypothetical
